# New Edges of RNA Adenosine Methylation Modifications

**DOI:** 10.1016/j.gpb.2016.05.003

**Published:** 2016-05-30

**Authors:** Ye Wang, Guifang Jia

**Affiliations:** Synthetic and Functional Biomolecules Center, Beijing National Laboratory for Molecular Sciences, Key Laboratory of Bioorganic Chemistry and Molecular Engineering of Ministry of Education, College of Chemistry and Molecular Engineering, Peking University, Beijing 100871, China

Recently an article published in *Molecular Cell* reveals the mechanism of a nuclear *N*^6^-methyladenosine (m^6^A) reader, the YTH domain-containing protein 1 (YTHDC1), in regulating pre-mRNA splicing [1]. Meanwhile, two additional articles published in *Nature* and *Nature Chemical Biology* report the first transcriptome-wide maps of *N*^1^-methyladenosine (m^1^A) at high resolution, suggesting a functional role for m^1^A in translation regulation [2], [3].

## m^6^A reader YTHDC1 in pre-mRNA alternative splicing

m^6^A is the most abundant endogenous mRNA modification, which is conserved across archea, bacteria, and eukaryotes [4]. Nonetheless, the importance of m^6^A in mammals had been underappreciated for about 40 years until the discovery of its reversibility by an m^6^A demethylase—fat mass and obesity-associated protein (FTO) [5] in 2011. Ever since, the widespread regulatory roles of m^6^A have been unraveled through the transcriptome-wide mapping of m^6^A modification [6], [7], the characterization of the second m^6^A demethylase AlkB homolog 5 (ALKBH5) [8] and three subunits of m^6^A methyltransferase complex (methyltransferase like 3, METTL3; METTL14; and Wilms tumor 1 associated protein, WTAP) [9], [10], and the functional studies of m^6^A readers YTH domain family protein 1 (YTHDF1) and YTHDF2 in humans, which regulates m^6^A methylated RNA stability [11] and translational efficiency [12], respectively. In addition, m^6^A in primary microRNAs can be recognized by another m^6^A reader, the heterogeneous nuclear ribonucleoproteins A2/B1 (HNRNPA2B1), which consequently recruits DiGeorge syndrome critical region 8 (DGCR8) and DROSHA complex and promotes the maturation of microRNAs [13], [14].

YTHDC1, as reflected by its name, contains the YTH domain that selectively binds to m^6^A [15]. Unlike the other two cytoplasmic m^6^A binding proteins YTHDF1 and YTHDF2, YTHDC1 is localized in YT bodies near the nuclear speckles [16], supporting its association with pre-mRNA splicing. Xiao and colleagues [1] identified several YTHDC1 partners including five *trans*-acting splicing factors (serine/arginine-rich splicing factors; SRSF1/3/9/7/10) by tandem-affinity purification following by mass spectrometric analysis, suggesting the potential regulatory role of YTHDC1 in pre-mRNA splicing. To test such possibility, they measured the alternative splicing (AS) events using RNA-seq data upon knockdown of YTHDC1 and its potential SRSF partners in HeLa cells, respectively. Their findings indicate that YTHDC1 and SRSF3 facilitate exon inclusion, while SRSF10 promotes exon skipping; however, silencing of other SRSF proteins (SRSF1, SRSF7, and SRSF9) has no significant effect on AS events. Photoactivatable ribonucleoside crosslinking and immunoprecipitation (PAR-CLIP) sequencing shows that the targeted regions of YTHDC1, SRSF3, and SRSF10 are enriched in the coding sequences (CDS) and the 3′ untranslated regions (UTR). Through analyzing the targeted exons, they further confirmed the opposite roles of YTHDC1/SRSF3 and SRSF10 in AS regulation. The change of AS events on the transcripts targeted by both YTHDC1 and SRSF3 in HeLa cells with YTHDC1 or SRSF3 silenced shows similar features with that in METTL3-silenced HeLa cells, suggesting that YTHDC1 and SRSF3 co-regulates AS events in an m^6^A-dependent manner.

Next, the authors set out to validate the interaction of YTHDC1 with either SRSF3 or SRSF10. PAR-CLIP data show that the YTHDC1 target regions are located closer to the binding sites of SRSF3 than those of SRSF10. *In vivo* and *in vitro* co-immunoprecipitation assay verifies that YTHDC1 directly interacts with SRSF3 and SRSF10 through the N-terminal of YTHDC1 and C-terminal of SRSF3 or SRSF10. The different AS events affected by YTHDC1/SRSF3 and SRSF10 prompts them to speculate that SRSF3 and SRSF10 might competitively bind to YTHDC1. Indeed they confirm the hypothesis using competing pull-down assays. The authors then examine whether YTHDC1 regulates localization of SRSF3 and SRSF10. Immunostaining assays show that silencing of YTHDC1 reduces SRSF3 but increases SRSF10 in nuclear speckle. Interestingly, this phenomenon can be rescued by complementation of wild-type YTHDC1, but not YTHDC1 mutant without m^6^A binding ability, indicating that YTHDC1 regulates the subcellular localization of SRSF3 and SRSF10 in an m^6^A-dependent manner. Further RNA binding assay shows that YTHDC1 deficiency disrupts the RNA binding of SRSF3 but enhances that of SRSF10, which can be complemented by wild-type YTHDC1, but not an m^6^A-binding-defective variant. These results indicate that the impact of YTHDC1 on AS events relies on the presence of m^6^A and the binding ability of YTHDC1 to methylated RNA.

**Figure 1:**
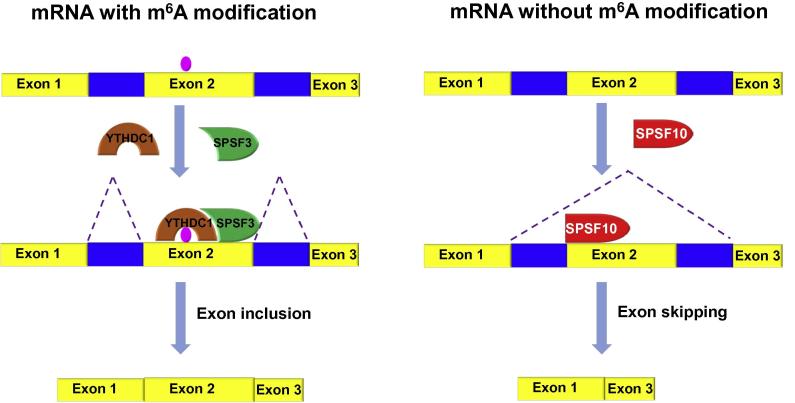
**A proposed model of pre-mRNA splicing regulated by YTHDC1** Under the conditions that m^6^A in pre-mRNA is recognized by YTHDC1, YTHDC1 recruits SRSF3 to promote exon inclusion; under the conditions that pre-mRNA does not contain m^6^A or pre-mRNA with m^6^A is not bound by YTHDC1, SRSF10 facilitates exon skipping. YTHDC1, YTH domain-containing protein 1; SRSF, serine/arginine-rich splicing factor.

Clearly, the comprehensive analysis presented by Xiao et al. reveals that m^6^A reader YTHDC1 facilitates exon inclusion by recruiting RNA splicing factor SRSF3 but blocking SRSF10 for its access to the binding regions of its target mRNAs (Figure 1). Indeed, apart from YTHDC1, m^6^A reader HNRNPA2B1 [14] and indirect m^6^A reader HNRNPC [17] are both involved in RNA splicing. What roles do these proteins play in AS? Are there any other splicing factors regulated by m^6^A? Does YTHDC1 play other regulatory role apart from splicing? These questions warrant further investigations.

## The reversible and dynamic m^1^A methylome in eukaryotic mRNA

m^1^A, another RNA adenosine methylation modification, has been identified in total RNA [18], rRNA [19], and tRNA [20] for decades. m^1^A modification contains a methyl group on *N*^1^ (hydrogen bond receptor) to form the positive charge and disturbs Watson–Crick base pairs. Unlike m^6^A, m^1^A can cause both reverse transcription stops and read-throughs accompanied by mismatches. m^1^A has been shown to affect the structure and function of tRNA and rRNA [21], [22]. However, the presence and functions of m^1^A in mRNA remain unknown.

**Figure 2:**
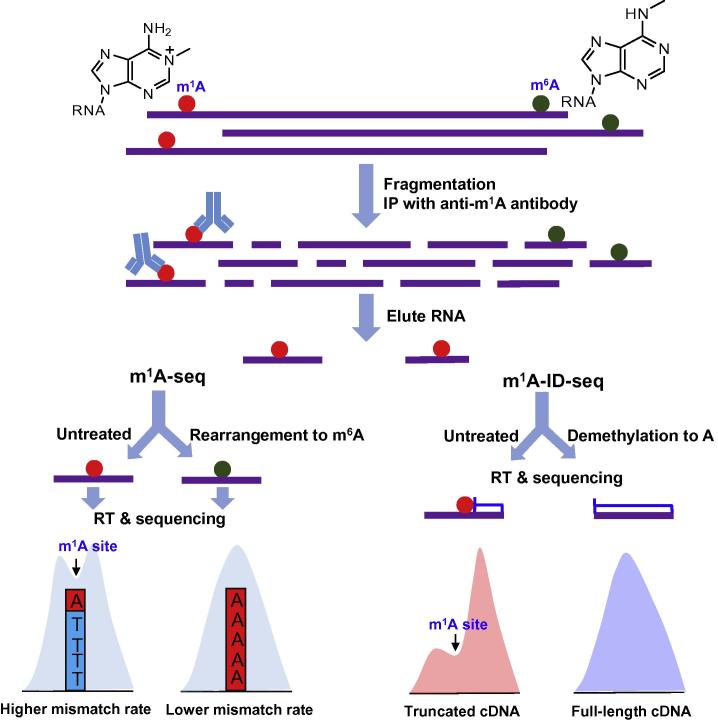
**Schematic outline of m^1^A-seq and m^1^A-ID-seq** In m^1^A-seq, mismatch rates caused by m^1^A (untreated sample) and m^6^A (chemical rearrangement) were compared. In m^1^A-ID-seq, cDNA truncations conferred by m^1^A (untreated sample) were compared to full-length cDNA (demethylation to A). IP, immunoprecipitation; RT, reverse transcription.

In the two recently-released papers, Dominissini et al. [2] and Li et al. [3] reported two transcriptome-wide sequencing methods (termed m^1^A-seq and m^1^A-ID-seq, respectively) to map m^1^A in mRNA at high resolution (Figure 2). Their work reveals that m^1^A is the second reversible and dynamic modification in eukaryotic mRNA. They firstly enrich m^1^A-containing mRNA fragments from human or mouse cell lines by m^1^A-specific antibody immunoprecipitation, and then take advantage of m^1^A property in reverse transcription to improve the sequencing resolution, albeit later on the two groups employ different approaches for locating m^1^A sites (Figure 2). As m^1^A modification can be converted to m^6^A in alkaline conditions (Dimroth rearrangement), Dominissini et al. treated a portion of precipitated m^1^A-containing mRNA fragments with alkaline buffer to chemically rearrange m^1^A to m^6^A prior to cDNA synthesis. By comparing mismatch rates between treated and untreated samples, they located m^1^A position within m^1^A peaks, in which mutation rates are high in the treated sample but low in the untreated sample. In this way, they can achieve m^1^A sequencing peaks at the resolution of 5–15 nucleotides (conserved m^1^A sites in rRNA can be mapped at the resolution of one nucleotide) [2] (Figure 2). Different from Dominissini et al., Li et al. used *Escherichia coli* AlkB protein to demethylate m^1^A to regular adenosine and performed cDNA synthesis with AMV reverse transcriptase to maximally confer cDNA truncations near m^1^A sites. In this way, they achieved the m^1^A map at the resolution of 55 nucleotides by comparing the m^1^A peak features between the untreated and treated samples [3] (Figure 2). In fact, both strategies, based on mutations or truncations, sacrifice the sequencing signal and lose some sequence information near the modified sites, which make it difficult to obtain single-base resolution m^1^A maps of high quality.

The relative abundance of m^1^A in mammalian mRNA is much lower (m^1^A/A: 0.015%–0.054% in cell lines and up to 0.16% in tissues) than that of m^6^A (m^6^A/A: 0.4%–0.6%) [2]. m^1^A-seq identified 7154 m^1^A peaks covering 4151 coding and 63 non-coding genes in humans [2], whereas m^1^A-ID-seq detected 901 m^1^A peaks with high confidence in 600 human genes [3]. Both studies show that most of the identified transcripts contain only one m^1^A peak. Unlike m^6^A peaks that are enriched in the last transcribed exon [6], [7], [23], [24], m^1^A peaks are highly enriched within 5’ UTR and near start codons.

According to the estimation of Dominissini and colleagues [2], ∼20% genes contain a single m^1^A. Through the deep analysis, they find that m^1^A is associated with canonical and alternative translation initiation sites, as well as the first splice site. Therefore they presume that the first spicing reaction might guide m^1^A deposition. m^1^A prefers more structured regions with high GC content and low minimum free energy. It is of note that m^1^A level and distribution pattern in mouse embryonic fibroblasts (MEFs) and mouse embryonic stem cells (mESCs) are comparable to those in human cell lines, suggesting an evolutionarily-conserved pattern of m^1^A methylome. They also survey the influence of different stress conditions on m^1^A, and find that the total level and peak number of m^1^A can be reduced by glucose starvation but enhanced by heat shock, indicating the dynamic feature of m^1^A under different physiological conditions. Given the close association of m^1^A with the translation initiation sites, Dominissini and colleagues examine whether m^1^A affects mRNA translation by using published ribosome profiling and proteomics data. Notably, m^1^A-containing genes have higher translation efficiency and protein levels compared to non-m^1^A-containing genes, implying that m^1^A modification is correlated with elevated translation.

Meanwhile, Li and colleagues [3] studied the m^1^A dynamics induced by H_2_O_2_ treatment and serum starvation. They propose that m^1^A may reside in a prominent motif with a GA-rich consensus. Similar with the aforementioned *Nature* paper, they state that m^1^A prefers structured sequences with high GC content. It is notable that ALKBH3 (human ortholog of *E. coli* AlkB) is found to be able to demethylate m^1^A in human mRNA, indicating that m^1^A is a reversible modification and may play an important regulatory role on mRNA.

Collectively, the two studies by Dominissini and his colleagues [2] and Li and his colleagues [3] provide the first map of transcriptome-wide m^1^A methylome and suggest new roles for m^1^A: this reversible modification is enriched around start codon, dynamically regulated by stress conditions, and correlated with elevated translation. Although the two m^1^A-seq techniques discussed here provide m^1^A maps with relatively-high resolution compared to m^6^A-seq method (at the resolution of ∼200 nucleotides), a big challenge is to develop single-base resolution methods for m^6^A and for m^1^A as well. Another challenge is to uncover the broader biological functions of m^6^A and m^1^A modifications. Future studies will focus on the identification and characterization of writer and reader proteins and functional roles of these two modifications. Given that m^6^A as an RNA structure switch affects RNA–protein interaction [17], the RNA structure changed by m^1^A modification might also play certain functions. We expect more investigations to draw a more comprehensive picture of RNA modification story.

## Competing interests

The authors declare that they have no competing interests.
